# Trust and compliance: Milieu-specific differences in social cohesion during the COVID-19 pandemic in Germany

**DOI:** 10.3389/fsoc.2022.989831

**Published:** 2022-12-21

**Authors:** Tim Schröder, Anne Speer, Patrick Sachweh, Olaf Groh-Samberg

**Affiliations:** Research Institute Social Cohesion (RISC), University of Bremen, Bremen, Germany

**Keywords:** social milieus, social cohesion, social integration, trust, conformity, COVID-19, socioeconomic status, basic human values

## Abstract

As a response to the COVID-19 pandemic, an increase in social cohesion was observed during the first wave and its aftermath. A closer look reveals heterogeneous responses regarding aspects of cohesion—such as trust in others and compliance with containment measures—that differ by individual socioeconomic and cultural characteristics. How these characteristics affect social cohesion in combination is rarely investigated. Therefore, we introduce the concept of social milieus, which addresses the interrelation of socioeconomic and cultural characteristics on the level of social groups, into the international debate. While previous studies have applied this concept to the analysis of social cohesion during the pandemic, they exhibit theoretical and empirical shortcomings. Hence, we develop a new theoretical model of social milieus and an empirical typology using the German sample of the European Social Survey. This typology is matched with data from the Research Institute Social Cohesion (RISC) for a milieu-specific analysis of social cohesion. Results show considerable heterogeneity in social cohesion during the first wave of the pandemic in Germany. Three social milieus with potentially conflicting modes of social cohesion regarding trust and compliance stand out while other milieus are less diverging as presumed in the literature. These modes can be interpreted as emerging from a combination of the milieus' socioeconomic position and basic human values. Thus, the new theoretical model and empirical typology of social milieus contribute to the understanding of how social cohesion has been contested between social milieus early in the pandemic.

## Introduction

After the outbreak of the COVID-19 pandemic and the issuing of the first lockdown measures in Germany, appeals to social cohesion and solidarity were frequent. Initially, between the first two waves of the COVID-19 pandemic in Germany, which peaked in April and November 2020, perceived social cohesion and interpersonal trust increased. This finding has been interpreted as an emotionally driven “rally-round-the-flag,” a short-term response of closing ranks in the face of an external threat (Bol et al., 2021). As the crisis progressed, however, analyses focusing on the over-time trend of responses to the COVID-19 pandemic showed that both institutional trust in the government and public health services as well as compliance with governmental recommendations (e.g., social distancing) decreased. In turn, concerns about social cohesion and the long-term consequences of restrictions, and the willingness among the non-vaccinated to participate in protests have increased (Frei et al., 2021; Grande et al., 2021)^1^. A closer look at the “rally” phase reveals that heterogeneity in institutional trust, attitudes toward political containment measures, and health concerns were already observed back then. Therefore, we suppose it is crucial to go beyond the prevailing focus on general trends within the German population and scrutinize group-specific heterogeneity in the perceptions of and responses to the pandemic and its political consequences in greater detail.

We suggest that a perspective focusing on “social milieus” holds promising insights for such a subgroup analysis. Social milieus can be defined as large latent groups sharing basic socioeconomic and cultural characteristics that are meaningful to their members, thereby shaping attitudes and (inter-)actions. We assume that the constitutive features of social milieus shape social cohesion in the face of the pandemic. Recently, various typologies of social milieus have been employed to analyze group differences in social cohesion during the COVID-19 pandemic (Sinus^®^ Institute., 2020; Beckmann and Schönauer, 2021; El-Menouar, 2021). However, all typologies have considerable limitations regarding conceptualizing cultural values, treatment of socioeconomic characteristics, or overall replicability (Sachweh, 2021). Moreover, the theoretical understanding of social cohesion in relation to social milieus is limited. While extant analyses point out heterogeneity in cohesion between milieus, they do not specify which (latent) social conflicts might emerge from milieu-specific differences in socioeconomic positions and cultural values. A theoretically founded and replicable typology of social milieus is needed to appropriately analyze social cohesion during the COVID-19 pandemic from the perspective of social milieus.

In this paper, we first discuss and define the concepts of social cohesion and social milieus. We then review recent findings on social cohesion during the first wave of the COVID-19 pandemic and its aftermath in general and between social milieus in particular, as revealed in previous typologies. Next, we propose a novel typology of nine social milieus in Germany based on Latent Class Analysis and data from the German sample of the European Social Survey (ESS) 2016 (*n* = 2,852). This typology overcomes the drawbacks of previous approaches as it is theoretically founded and replicable with publicly accessible large-scale survey data. We apply this typology of social milieus to explore intergroup differences in two relevant aspects of social cohesion during the first wave of the COVID-19 pandemic and its aftermath: trust in social cohesion and concerned compliance with restrictions. The empirical analysis is based on the German pilot study of the Research Institute Social Cohesion (RISC), which was conducted from April to September 2020 and can be matched with the ESS data (*n* = 589). Our typology of social milieus allows a closer assessment of different modes of social cohesion during the COVID-19 pandemic and the potentially conflictual relations between milieus along the lines of stratification and values.

## Theoretical background

### The concept of social cohesion

The meaning of the concept of social cohesion differs considerably within the scientific literature (Chan et al., 2006; Schiefer and van der Noll, 2017)^2^. Moreover, social cohesion is used vaguely in ordinary language, and broader “conceptions” according to the idea of a “good society” are attached to this term in public opinion and scientific discourse—putting the term in danger of becoming an “empty signifier” with a normative character (Deitelhoff et al., 2020, p. 13). The presence of different conceptions highlights that social cohesion occurs in various forms that may differ between social groups. Therefore, the task is to find an analytical definition of social cohesion on the societal level close to everyday use, minimal in scope, and at the same time suited to analyze different group-specific conceptions.

Within the past years, different concepts of social cohesion on the societal level have been developed, that aim to address the above features. Chan and Goldthorpe (2004, p. 290) define cohesion as “a state of affairs concerning […] interactions among members of society as characterized by a set of attitudes and norms that includes trust, a sense of belonging and the willingness to participate and help, as well as their behavioral manifestations.” This concept is designed for cross-cultural and historical comparison, reducing social cohesion to its supposed smallest common denominator and a gradational “more-or-less” logic. However, a gradational understanding of cohesion is ill-suited to capture qualitatively different forms of cohesion. Furthermore, a focus on interactions or a sense of belonging risks inserting a bias toward a specific “communal” (“gemeinschaftlich”) form of cohesion at the group level (Stanley, 2003, p. 10). Other definitions are oriented toward the macro-level and expand the cohesion concept by a “modern,” pluralistic type, thereby deliberately following a normative interpretation. For example, the “Social Cohesion Radar” defines a “cohesive society” by three domains: (a) “resilient social relations” (including interpersonal trust and acceptance of diversity), (b) “a positive emotional connectedness between the community and its members” (e.g., identification) and (c) “a pronounced focus on the common good” (e.g., civic participation) (Dragolov et al., 2016, p. 1). This approach, again, follows a gradational logic by building a single formative index score. Hence, different types of social cohesion are not distinguished.

Grunow et al. (2022) have proposed using the concept of “social integration”, which is similar to cohesion but systematically rooted in theoretical debates in sociology. While cohesion refers to a group property, Grunow et al. conceptualize integration as a multi-level concept referring to the “inclusion” of actors into social orders from interactions to social groups to societal subsystems (Luhmann, 1997, p. 619). The social integration of individuals into society at large results from their multiple inclusions into various nested, neighboring, or intersecting social orders below the societal level. Grunow et al. (2022) identify four basic ingredients of social integration: (1) *Consensus* as shared conceptions of the given, desirable, or normatively required; (2) *Trust* in fellow citizens to adhere to rules; (3) *Conformity* with various kinds of norms, customs or traditions; (4) *Cooperation* with others. Social integration is not conceptualized as the maximization of all ingredients within a more-or-less logic but as a well-balanced mid-point on a continuum ranging from disintegration on the one hand to over-integration on the other hand. Importantly, it is not the addition but the interplay of the four ingredients that generates social integration. This inherent multi-dimensionality allows for group-specific, substantially different conceptions of social cohesion as a group property to emerge from various combinations of the ingredients. This reflects Durkheim (1897[2002]) central insight that social integration is not a matter of degree but types. Conflicts between groups about the desirable mode of social cohesion play a significant role in pluralistic democracies, connecting antagonistic groups (e.g., parties in collective bargaining) instead of segregating them (Coser, 1956[1964]; Lipset and Rokkan, 1967).

*In light of the above discussion, we suggest using the term “social integration” as an overarching multi-level concept, whereas “social cohesion” refers to the internal integration of social groups through specific constellations of consensus, trust, conformity, and cooperation. This distinction allows us to identify social groups and their differences, to relate them on the societal level, and thus assess potential social conflicts*.

The COVID-19 pandemic poses a particular context in which issues of group-specific social cohesion are contested with regard to overall social integration on the societal level. Governments impose measures, and people depend more than before on the actions of others. This makes compliance and trust very salient issues. Compliance with measures can be seen as a manifestation of the conformity ingredient of social cohesion. Trust that others comply and trust in society's capability to handle the virus reflect a manifestation of the trust ingredient. Thus, in this paper, we focus on trust in social cohesion and concerned compliance with governmental measures—short: trust and concerned compliance—as two highly relevant contextual manifestations of social cohesion during the first phase of the COVID-19 pandemic.

### The concept of social milieus

Durkheim (1895[1982], 1897[2002]), introduced the notion of “social milieus” into sociology for capturing emergent, intermediate, and large social groups that contribute to the integration of individuals into society. Yet, except for France and Germany, the term “social milieu” has not become well-established in the international sociological debate^3^. Instead, “social class” prevails, with social stratification as its main characteristic. However, this concept is ambiguous and contested. The debate, for example, if occupational class schemes can still explain political behavior, is ongoing (Dalton and Klingemann, 2013; Evans and Langsæther, 2021). Undisputed is the observation that cultural issues beyond socioeconomic interests, like post-materialistic values, have become more salient. *Cultural class analysis* has emerged as a new perspective on classes in the tradition of Bourdieu, acknowledging the importance of socioeconomic inequality *and* culture (Vester, 2013; Savage, 2021). *To avoid the ambiguity of the “cultural class” terminology and address the interrelation of socioeconomic and cultural aspects in constituting large social groups with specific modes of social cohesion, we use the term “social milieus.”*

**Figure 1:**
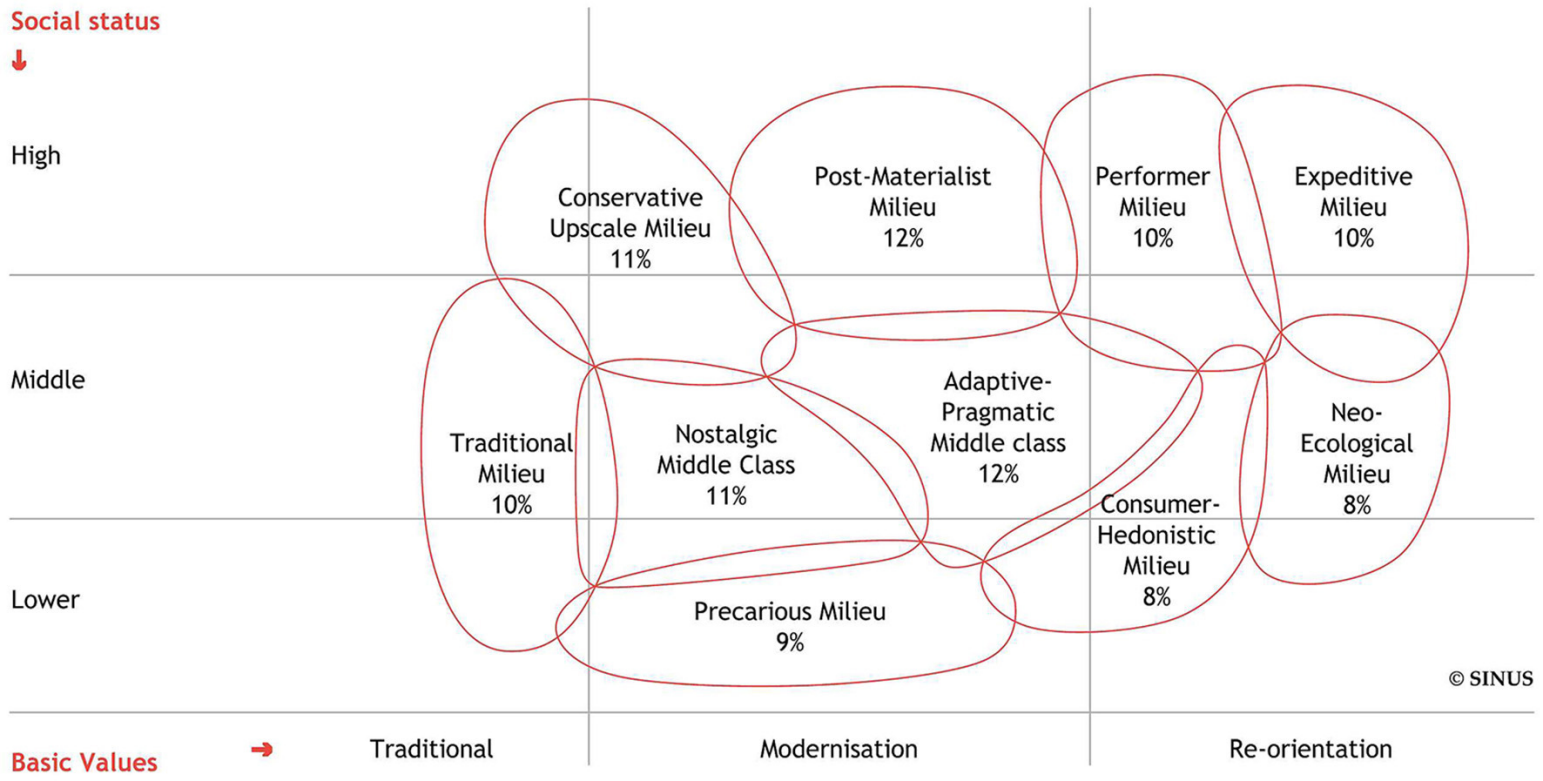
Sinus^®^ milieus. Source: Sinus^®^ Institute. (2021).

In the past couple of years, theoretical milieu conceptions and empirical milieu studies have been developed to analyze social cohesion. The most elaborate theorization of social milieus has been developed by Vester et al. (1993[2001]). Empirically, it was based on the widely used Sinus^®^ milieus (for a more comprehensive overview, see Groh-Samberg (forthcoming)). The Sinus^®^ milieu typology, established in the 1970s, serves to map relevant patterns of the social structure” and also of society-wide “cleavages” (Flaig et al., 1994, p. 43f). Hence, milieus are interpreted as (real) lifeworlds of large groups of individuals. Originally grounded in qualitative explorations (Flaig et al., 1994), the typology was validated quantitatively through cluster analyses of indicators measuring value orientations and life goals. *Post-hoc*, it was revealed that they also “produced” vertical stratification by education and income (Flaig et al., 1994, p. 49, 70). Eventually, the milieu typology was depicted on two axes: a vertical axis is divided into lower-, middle-, and upper-class strata. A horizontal axis ranges from traditional values of conservation, security, and conformity to reorientation values oriented toward openness to change and exploration of new lifestyles. The latest version identifies ten milieus in Germany (see Figure 1). The usefulness of the Sinus^®^ milieu typology has been demonstrated in various fields in the social sciences, such as political culture (Flaig et al., 1994).

Vester et al. (1993[2001]) deliver a theoretical interpretation of the Sinus^®^ milieu typology based on Bourdieu (1979[1984]), which they explicitly developed for the analysis of social integration. Social milieus are characterized in terms of a specific “habitus”: the attitude pattern of an individual, expressed in taste, mentality, and a particular ethic of the conduct of everyday life (Vester et al., 1993[2001], p. 25). Milieus are thus defined as “groups with similar habitus, coming together through kinship or neighborhood, work or education and develop a similar everyday culture. They are connected through social cohesion or only through similar orientation of habitus” (Vester et al., 1993[2001], p. 24f, own translation). Following Bourdieu, the importance of the socioeconomic status axis of the Sinus^®^ milieu typology is particularly emphasized. Between the three strata, two dividing lines are identified: The boundary of “respectability” separates the “decent” middle class from the “undeserving” lower class, and a boundary of (cultural) “distinction” separates the upper class from the middle and lower classes (Vester et al., 1993[2001], p. 26ff, own translation). Finally, Vester et al. (1993[2001], p. 427ff) also provide a detailed empirical account of various modes of social cohesion. In particular, the theoretical foundation of the socioeconomic axis allows for the integration of potential conflicts over resources into the milieu approach—a feature currently pronounced in the face of the perceived threats to social cohesion (Hradil, 2022).

Yet, the socioeconomic dimension is not a constitutive part of the empirical Sinus^®^ milieu typology, and the conceptualization of cultural values follows a unidimensional logic, as it only contains a modernization axis. Current value theories identify at least one more value dimension (Inglehart and Welzel, 2005). Schwartz's (2012) comprehensive approach to values identified a second value dimension ranging from self-enhancement (power and achievement) to self-transcendence (universalism and benevolence) values (see Miles, 2015). This dimension is of considerable importance in contemporary debate. For example, Reckwitz (2019) identifies an “old middle class” composed of intermediate education and supporting self-enhancement values, opposing a “new middle class” with higher education and self-transcendence values in Germany. Moreover, he supports a milieu differentiation of the middle class according to the Sinus^®^ typology, even though it does not account for self-enhancement and self-transcendence values. In the context of the COVID-19 pandemic, the importance of these values is especially pronounced as it can be expected that governmental measures to contain the virus go against motives of self-enhancement. Another disadvantage of the Sinus^®^ typology is that the Sinus^®^ institute does not reveal the clustering algorithm of the milieus, making proper scientific research difficult (Sachweh, 2021). A replicable empirical milieu typology with a comprehensive conceptualization of cultural values and an appropriate consideration of socioeconomic characteristics is still lacking. This paper's empirical part builds on a new milieu model that fits these criteria.

The definition of social milieus by Vester et al. has two implications: first, the socioeconomic and cultural dimensions are equally important. Second, a common habitus is sufficient for milieus to exist; a milieu consciousness is not a necessary characteristic. We follow these considerations and take up the concepts of cohesion and integration defined above. We define *social milieus as large, latent social groups composed of socioeconomic and cultural components. Their specific compositions result in respective modes of social cohesion. These modes integrate individuals into society differently and stand in (potential) conflict with each other. Thus, social milieus serve as a touchstone for social integration and social cohesion during a crisis like the COVID-19 pandemic*. Before turning to our milieu model, we first document empirical findings on social cohesion during the COVID-19 pandemic and how these relate to existing milieus approaches.

## State of research

### Social cohesion during the COVID-19 pandemic

In the first two waves of the pandemic, which peaked around April and November 2020, respectively, perceived social cohesion and institutional trust within the German population have increased compared to the times before the pandemic (Kühne et al., 2020; Delhey et al., 2021). This finding is consistent with the “rally-around-the-flag” thesis. The levels of interpersonal trust during the first wave of the pandemic were shown to remain stable (Delhey et al., 2021) or increase (Adriaans et al., 2021) compared to before the pandemic. Moreover, trust in the government's ability to avoid unequal treatment of different social groups was high (Busemeyer, 2020, p. 1). Trust, in turn, served as a precondition for compliance with measures (Bargain and Aminjonov, 2020).

Compliance with protective recommendations has slightly decreased between the first two waves (Adriaans et al., 2021). During this period, the willingness to get vaccinated if vaccination would be enforced by law was relatively low and further decreased over time (Schmelz and Bowles, 2021). Early in the pandemic, several political measures like social distancing rules, compulsory masks, and cancellation of events were widely supported. In contrast, the attitudes toward other actions, like the shutdown of public institutions (e.g., daycare facilities) or a possible mandatory vaccination, were polarized (Beckmann and Schönauer, 2021).

When looking beyond population averages, heterogeneity is revealed. For compliance with measures, a stable center of the population and no polarization between large groups could be observed. Instead, the margins were somewhat eroding as skeptics became more radicalized (Busemeyer et al., 2021b), eventually turning into a social movement of Corona protesters, the so-called “Querdenker” (see also Frei et al., 2021; Grande et al., 2021). While these protesters over-proportionally voted for the Greens and the Left party in the past, during the pandemic, many switched to the COVID-19 protest party “die Basis” or the right-wing populist AfD (“Alternative for Germany”).

Heterogeneity also shows when social groups are differentiated by socioeconomic and cultural dimensions. For instance, those with low education or low incomes suffered not only additional income losses (WSI, 2020) but also perceived social cohesion to be more endangered (Brand et al., 2021) and were more prone to endorse conspiracy beliefs regarding vaccination (Jensen et al., 2021). In the cultural dimension of attitudes, values, and social identities, “initial national or global unity” turned into “rivalrous cohesion” between groups in later stages (Abrams et al., 2021, p. 201, 205). These conflicts revolve around the free riding of groups who do not adhere to measures but benefit from public spending and collective compliance. They also involve moralism and strengthening the social identity of groups who do adhere to measures (Abrams et al., 2021, p. 204). Moreover, social cohesion is compatible with demarcation from or discrimination of ethnic groups due to the allegedly spreading of the virus (Dollmann and Kogan, 2020). Hence, it is crucial to identify heterogeneity: dominating and marginalized, vulnerable or radicalized social groups within society. What is still missing is an overall picture of these groups in relation to each other regarding social cohesion. Recently, three empirical milieu approaches aimed to carry out this task and analyze group-specific social cohesion during the COVID-19 pandemic.

### Social milieus and social cohesion during the COVID-19 pandemic

El-Menouar (2021) identifies seven “value milieus” through principal component and cluster analyses of Schwartz (1992) basic human values. Overall, during the second pandemic wave, there is considerable approval of the importance of protecting lives and, consequently, the requirement of policy measures that restrict liberty rights. The majority (80%) of the respondents approve of prioritizing the protection of life (El-Menouar, 2021, p. 25). However, mainly the individualistic materialist milieu, with a large proportion of older, self-employed individuals with higher incomes, points to the economically detrimental effects, thereby strongly disagreeing with the humble humanist milieu, which is academic and exhibits universalistic values. While the achievement-oriented milieu (also with high incomes) has a more conservative background than the individualistic materialists, for both milieus self-enhancement values are predominant. Consequently, they endorse the individual freedom of choice and oppose vaccination—thereby strongly differing from the humanist and (older) safety-oriented conservative milieus. In contrast to the rally thesis or, at least, in anticipation of future developments, a majority of 69% expect that the COVID-19 pandemic would polarize society. Here, too, considerable milieu heterogeneity is shown. For example, the achievement-oriented milieu expects a positive impact on social cohesion and has faith in overcoming the COVID-19 crisis. The materialists, in turn, disagree strongly but, at the same time, find that a profound societal change in the face of the pandemic is unnecessary. These milieu differences might result from different positions on the conservation vs. openness axis.

Beckmann and Schönauer (2021) use cluster analyses with data from an online survey collected in August and September 2020. They detect four social milieus composed of two factors extracted by factor analyses: (1) a factor comprising materialistic values and right-wing political orientation as opposed to post-materialistic values and left orientation, and (2) a socioeconomic factor composed of income, education, and class self-placement. The resulting left-liberal intellectual milieu and the (right-wing) conservative-established milieu have high positions on the socioeconomic dimension. In contrast, the (materialistic-right) traditional and the (postmaterialistic-left) alternative milieu are placed at the lower end. While more than 80% of the conservative milieu assessed the fight against the coronavirus positively, this applies to only 63% of the alternative milieu, the two other milieus lying in between. Concerning attitudes toward other issues, the restriction of migration, climate protection, and the reduction of social inequality, however, the left-liberal and alternative milieus resemble one another.

Finally, a third study conducted in May 2020 employs the Sinus^®^ milieus to analyze social cohesion (Sinus^®^ Institute., 2020). The liberal-intellectual or post-materialist milieu as the “guiding milieu” (“Leitmilieu”) with the highest amount of resources and moderate modernization orientation take the threat posed by the coronavirus seriously and was satisfied with the (extent of) governmental actions. This milieu is, to a relatively low extent, concerned about the effects of the pandemic on democracy and personal freedom and instead expects a positive impact. The precarious milieu stands in stark contrast to this milieu: the governmental actions are evaluated negatively and as too far-reaching, and the members of the milieu feel irritated and are worried about the negative impact of the pandemic on democracy and personal freedom. Other milieus stand between the liberal-intellectual/post-materialist and the precarious milieu regarding specific indicators. For example, the traditional and adaptive-pragmatic middle-class milieus do not consider the coronavirus as threatening. The latter assesses mandatory face masks negatively. The nostalgic middle class assesses the governmental measures as too far-reaching but prefers health over the economy when asked about the duration of measures, while the performer and the expeditive milieus put the economy first.

The three milieu approaches detect heterogeneity between groups and find certain milieus that oppose each other (conservative vs. alternative, liberal vs. precarious). However, every conceptualization has its theoretical or empirical deficits: El-Menouar (2021) value milieus do not contain a stratification dimension, and the value dimension used by Beckmann and Schönauer (2021) is unidimensional and mixes up general values with particular political attitudes. The inadequacies of the Sinus^®^ milieus have already been addressed in section “The concept of social milieus” Overall, all conceptions miss a closer assessment of the different modes of cohesion and the potentially conflictual relations between milieus along socio-economic or cultural lines.

### A new model of social milieus

We developed a theoretical model of social milieus as an attempt to overcome these shortcomings (Groh-Samberg (forthcoming)). The model carries forward the conceptual work of Vester et al. and is empirically replicable with publicly accessible large-scale data. Above all, a socioeconomic and a cultural dimension are distinguished. These dimensions are assumed to produce potentially conflicting modes of social cohesion and related practices.

**Figure 2:**
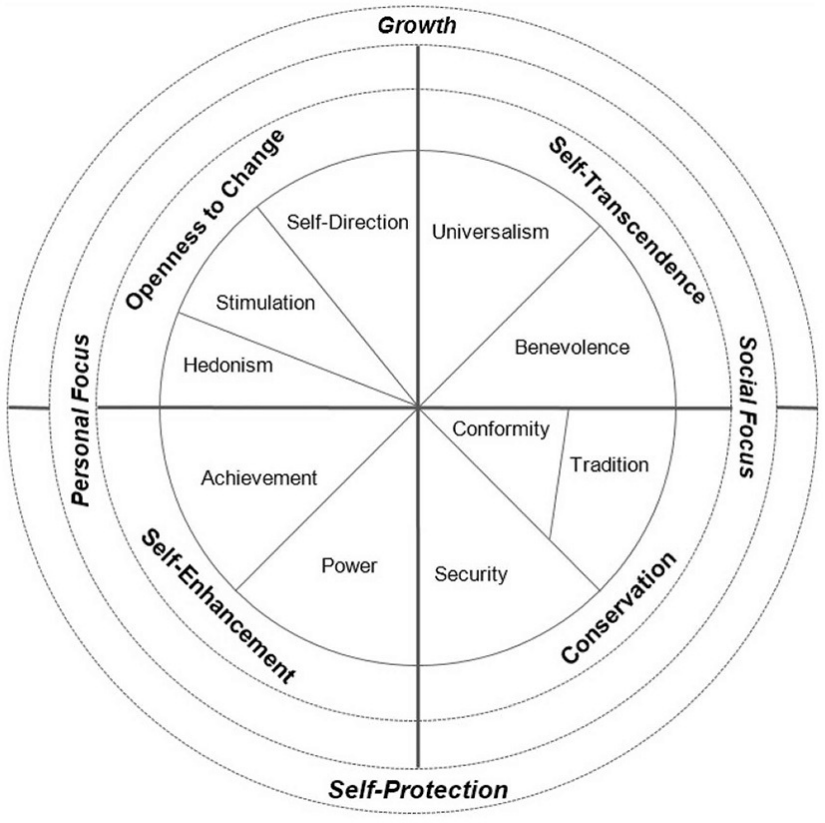
Schwartz's basic human values. Source: Magun et al. (2016).

We conceptualize the socioeconomic dimension as involving resources, which shape life chances and are recognized as such. As a first empirically tractable approximation, we include the level of formal education and household income as central indicators of socioeconomic status (Ganzeboom et al., 1992). In addressing the cultural dimension, we build on the concept of values, which has been revived in sociology and recognizes the role of actors as well as conflictual relations between social groups (Miles, 2015). Values are considered to be part of an individual's socially shaped “mentality” (Geiger, 1932) or “habitus” (Vester et al., 1993[2001]; Longest et al., 2013) and guide social evaluations and actions. Similar value profiles across individuals can thus be seen as part of social milieus. We go beyond unidimensional conceptions of values and build on basic human values as theorized and tested by Schwartz (1992, 2012). Schwartz identifies ten basic human values that can be arranged in a circumplex structure in which adjacent values are compatible with each other, and opposite values stand in (potential) conflict (see Figure 2). These values can be condensed to four higher-order values that can be organized along two axes ranging from self-transcendence (e.g., universalism) to self-enhancement (e.g., achievement) and from openness (e.g., self-direction) to conservation (e.g., tradition). Finally, based on the endorsement of each of two adjacent higher-order values, four value foci can be identified: a growth focus (openness and self-transcendence), a social focus (self-transcendence and conservation), a self-protection focus (conservation and self-enhancement), and a personal focus (self-enhancement and openness).

As has been said, the literature is ambivalent about the interrelation of the two milieu dimensions of socioeconomic position and culture. A major advantage of the concept of social milieus is that the role of stratification and culture in shaping large latent groups can be assessed empirically (Chan and Goldthorpe, 2004; Vester, 2013). Some milieus might be determined by very specific value profiles, thereby spanning over a broader range of socioeconomic positions, while other milieus might be more strongly characterized by their socioeconomic position. The only assumption we make is that values and socioeconomic indicators are not scattered freely over the entire range of the two-dimensional space but rather clustered in specific formations, resulting in a small number of large latent social groups of different sizes within society, i.e., social milieus. Empirically, in Germany, education and income are positively correlated with self-transcendence- and self-enhancement values (Meuleman et al., 2012). Education was furthermore positively correlated with openness and negatively correlated with conservation.

How do social milieus differ concerning social cohesion? As social milieus are defined by their socioeconomic position and cultural values, a brief look at the relationship between these indicators and trust and compliance as highly relevant aspects of social cohesion during the COVID-19 pandemic is worthwhile. The socioeconomic position is found positively related to interpersonal and institutional trust in general (Kim et al., 2022) and an increase in general trust, specifically during the COVID-19 pandemic (Wu et al., 2022). Findings on the relation of socioeconomic status with compliance are rather mixed: positive and negative associations were found (Nivette et al., 2021; Lee et al., 2022). Regarding values, self-transcendence is positively related to generalized interpersonal trust (Michalski, 2019) and compliance with governmental measures during the pandemic (Lake et al., 2021), and conservation (openness) values are positively (negatively) related to institutional trust and compliance (Pavlović Vinogradac et al., 2020; Bonetto et al., 2021; Cajner Mraović et al., 2021). While these bivariate associations are informative, our multidimensional milieu typology allows us to analyze trust and compliance for groups with certain combinations of socioeconomic positions and cultural values.

Considering the theoretical milieu accounts (section “The concept of social milieus”) and previous empirical findings (sections “Social cohesion during the COVID-19 pandemic”, “Social milieus and social cohesion during the COVID-19 pandemic”, and the preceding paragraph), some general expectations for our milieu model can be derived. In accordance with the “rally” thesis, we expect most milieus to show high levels of trust and compliance. Yet, some milieus should deviate from this homogeneity in the early stage of the pandemic. We expect to find milieus similar to the established conservatives (Beckmann and Schönauer, 2021) and safety-oriented conservatives (El-Menouar, 2021). This means, in line with the bivariate findings reported above, that throughout all socioeconomic positions milieus with a conservative or social (conservation and self-transcendence) value focus have high levels of trust and compliance. Furthermore, we expect that milieus with a growth focus (self-transcendence and openness values) show high trust and compliance only when they also hold higher socioeconomic positions. Such milieus are part of Reckwitz (2019) “new middle class”: the liberal intellectual, performer, and expeditive milieus (Sinus^®^ Institute., 2020) and the humble humanists (El-Menouar, 2021). In contrast, milieus that combine lower resources and high openness values should be associated with low trust and compliance, similar to the alternative milieu (Beckmann and Schönauer, 2021). In accordance with El-Menouar (2021) individualistic materialists, we expect to find at least one milieu with intermediate to higher socioeconomic status and a personal value focus (self-enhancement and openness values) that shows low levels of trust and compliance. Moreover, we expect to find at least one milieu that belongs to the “old middle class” and, according to Reckwitz (2019) has intermediate education, higher incomes, holds a protection value focus (self-enhancement and conservation), and thus resembles El-Menouar (2021) achievement-oriented milieu. For this (these) milieu(s) no consistent expectations about the mode of social cohesion can be derived. The protection value focus comprises two value dimensions with opposing associations with cohesion which may cancel out. Following Reckwitz's milieu differentiation, the old middle class should be approximately located on the socioeconomic and value dimensions near four Sinus^®^ Institute. (2020) milieus that were identified as differing in attitudes toward social cohesion: the established conservatives, the traditional milieu, the nostalgic middle class, and the adaptive-pragmatic middle class. Finally, we expect to find a precarious milieu (Sinus^®^ Institute., 2020) with no clear value focus but low socioeconomic resources and low levels of trust and compliance.

We emphasize that our milieu model goes beyond a variable-based analysis and captures whole value profiles of social milieus in combination with their socioeconomic positions. The model is suited, for instance, to uncover what El-Menouar (2021) could only suspect: that two milieus with similar value profiles have different modes of social cohesion due to different socioeconomic positions. Thus, our milieu approach allows for new comprehensive accounts of how value profiles and socioeconomic positions relate to social cohesion in the context of the COVID-19 pandemic.

## Materials and methods

In the empirical part of this paper, we first develop an empirical model of social milieus. In the next step, we analyze milieu differences in the two cohesion factors during the first wave of the COVID-19 pandemic and its aftermath: trust in social cohesion and concerned compliance with measures to contain the virus.

### Materials

We use the German subsample of the European Social Survey (ESS) Round 8 in 2016 (*n* = 2,852) to identify social milieus and handle missing values in the milieu indicators by listwise deletion (*n*= 2,470). To account for sample selection bias, nonresponse, noncoverage, and sampling error, we apply the ESS's post-stratification weight (including the design weight). To explore milieu-specific differences in manifestations of social cohesion during the COVID-19 pandemic, we use the RISC pilot study 2020. The RISC pilot study was designed as a pretest for the first wave of the RISC cohesion panel and conducted from April to September 2020, the peak of the first wave of the pandemic and its aftermath. It is a subsample of the German sample of the ESS 2016 and includes respondents who consented to participate in the RISC pilot study and also agreed to match their RISC data with the ESS8 (*n* = 589). The matching of the ESS8 with the RISC data allows linking social milieu membership with measures of trust in social cohesion and concerned compliance as responses to the COVID-19 pandemic. In the RISC data, respondents from East Germany and those with high education are overrepresented (see Supplementary Table S1). However, we refrain from using RISC sample weights to correct this bias. The weights are based on the full RISC sample (*n* = 868), a different sample that includes participants who did not agree to a matching with their ESS data and the participant's household members. Also, the standard errors of the weighted sample would be underestimated. Either way, the direction and significance of the effects do not change when weights are applied.

#### Identification of social milieus

As argued above, we conceptualize social milieus as constituted by a socioeconomic and a cultural dimension^4^. The socioeconomic dimension comprises income and education. Income was measured as total net household income quintiles. To make income comparable across households, it was equalized by dividing it by the square root of household size (OECD, 2020) and then categorized into five groups. Education was categorized into three groups: low (no degree, or lower secondary school, i.e. “Hauptschule”), intermediate (intermediate secondary school, i.e. “Realschule”), and high (upper secondary school, i.e. “Abitur” or “Fachhochschulreife”).

The cultural dimension of basic human values was measured by the 21-items *Portrait Value Questionnaire* (PVQ-21) (Schwartz et al., 2015). Here, descriptions of a fictional person were presented, and participants were asked to assess to what degree the fictional person is like them on a 6-point scale ranging from “very much like me” to “not like me at all.” An example item for self-transcendence is: “It is important to her/him to listen to people who are different from her/him. Even when she/he disagrees with them, she/he still wants to understand them.” As recommended by Schwartz (2020), the participant's responses to the 21 value items were person-centered (i.e., ipsatized: the within-person mean of all 21 items was subtracted from each value item) to deal with response bias and obtain the relative value priorities for each participant.

#### Trust in social cohesion and concerned compliance

Social cohesion during the COVID-19 pandemic was measured by seven statements and assessed on a 5-point scale ranging from “strongly disagree” to “strongly agree.” These items were selected on the grounds of face validity, and perceived relevance as no prior measure of such attitudes existed. Exploratory factor analysis with rotated and oblique factors (quartimin method in Stata^®^ 15) revealed two meaningful factors (see Supplementary Table S2). One factor can be denoted as “*trust in social cohesion*” (in short, “*trust*”) in the face of the COVID-19 pandemic. An item loading high on this factor (0.69) is: “The handling of the coronavirus shows that we can rely on “*gesellschaftlicher Zusammenhalt*” in our society.” The term “gesellschaftlicher Zusammenhalt” literally translates as societal holding together” and roughly as “social cohesion.” A second item is a negative rewording of this item (factor loading: −0.63). The third item (factor loading: 0.62) is worded: “I trust that the fellow citizens accept measures to contribute to containing the virus.” Although two items may involve institutions or collective actors as they are directed at the society at large, we rather interpret the factor as a measure of generalized interpersonal trust. The second factor was designated “*concerned compliance with measures*” (in short, “*concerned compliance*”). One item was worded, “I accept the restrictions to contribute my share to contain the virus” (loading: 0.56), and conveys compliance. While the second item (“I think that the measures to contain the coronavirus are excessive”) with a negative loading (−0.64) also refers to restrictions, the third item expresses concerns (“I am concerned about the spreading of the coronavirus,” loading: 0.52). Finally, one item loaded moderately on both the “trust” (.47) and “concerned compliance” (0.3) factors and captured institutional trust (“I trust that necessary measures are taken to contain the coronavirus”). The two factors were moderately correlated (*r* = 0.37). The factor scores for each respondent were predicted and saved for further analyses.

### Methods

#### Typology of social milieus

As has been elaborated in section The concept of social milieus, we follow the long-standing tradition of cultural class and milieu analysis that refers to “networks of statistical relations” (Bourdieu, 1979[1984]:103) and is based on the conviction that describing and comparing types is not a mundane task but a valid argument in its own right (Gerring, 2012). We use Latent Class Analysis (LCA) in Latent GOLD^®^ 6.0 (Vermunt and Magidson, 2021) to identify social milieus as a small number of large classes of individuals with similar characteristics on the two theoretically derived dimensions. LCA is an advancement of cluster analysis that is model-based (in the tradition of structural equation modeling) and allows for a probabilistic assignment of individuals to classes (Masyn, 2013; Savage et al., 2013). It is suited as a tool to identify large classes or milieus without excluding the empirical possibility of a gradational social structure (Grusky and Weeden, 2008). Moreover, it allows capturing both the socioeconomic positions and complete value profiles of social milieus simultaneously. This is a substantial advantage for the comprehensive analysis of values since the Schwartz values share meaningful variance. In variable-based regression analysis, adding two or more values would suppress the meaningful common variance of the values. Therefore, regression analysis is not able to adequately capture complete value profiles. Moreover, using regression analysis in an exploratory way, i.e., regressing all milieu indicators and their interactions on the outcomes introduces low statistical power due to the small sample size, an inflated chance of type-I errors, and considerable complexity. We thus used LCA as a powerful method to comprehensively capture milieu characteristics and reduce complexity by developing a theoretically informed multidimensional typology.

As described in detail in section “Identification of social milieus,” we use income, education, and the 21 person-centered basic human value items as indicators of the LCA^5^. We furthermore use four Bayesian priors that prevent model nonidentification without significantly changing the results (Vermunt and Magidson, 2016, p. 50). As an implication of this procedure, Posterior Mode estimation is applied instead of Maximum Likelihood. We use the Latent GOLD^®^ 6.0 default algorithms (Expectation Maximation in combination with Newton-Raphson) for maximizing the Log-Posterior function and run the model with 400 starting values to reach the global maximum with high certainty (see the Latent GOLD^®^ 6.0 syntax in the Supplementary material).

For deciding on the number of classes, we consult several information criteria and finally assess the candidates with a good fit based on theoretical grounds, as recommended by Nylund-Gibson and Choi (2018). According to our definition of social milieus, we inspect several solutions with an acceptable fit. The information criteria inform about the goodness of fit and are based on the Log-Posterior of the specific class solutions (Supplementary Table S3). The lower these information criteria, the better the model. The AIC and AIC3 penalize for the number of parameters and often produce solutions with a large number of classes in large samples. Since our sample is relatively large, we prefer the CAIC, BIC, and SABIC that additionally penalize for sample size (Vermunt and Magidson, 2016). The SABIC, however, penalizes sample size only to a very low extent and therefore did not reach a minimum within the class solutions up to 15 classes which we consider meaningfully interpretable. The CAIC and BIC reach a minimum at 13 and 14 classes, respectively^6^. Hence, we first inspect the 13-class and 14-class solutions closer, find that they are highly similar, and hence prefer the more parsimonious model. The relative fit improvement can additionally be consulted for finding the best class solution (Nylund-Gibson and Choi, 2018). It is high for 3, 6, 9, and 13 classes for all information criteria. Thus, we compare the 13-class to the 9-class solution. Overall, similar milieus are identified. The 13-class solution provides a more nuanced differentiation of the milieus. On the one hand, this reveals some heterogeneity in the upper and lower classes which is not visible in the 9-class solution. On the other hand, some smaller milieus within the middle class strongly resemble each other within the 13-class solution in terms of their socioeconomic and cultural characteristics. We finally choose the 9-class solution as the more parsimonious model, suited for analyzing the general milieu landscape. The 13-class solution might be consulted for more specific milieu differentiations in future research (see Supplementary Figure S1).

Beyond the chosen milieu model, we conducted robustness checks regarding validity and sensitivity^7^. Results only differed significantly when no person-centering was applied or when the person-centered values were further divided by the individual's standard deviation. We refrained from using these transformations. The former does not consider individual response styles, while the latter neglects meaningful individual differences in variances of value ratings (Schwartz, 2020). We also did not reduce the relatively high impact of the 21 value indicators on the milieu solution by using variable weights (Vermunt and Magidson, 2021). This procedure produced considerable side effects which have not been investigated well yet. Furthermore, the LCA was not based on factor or index scores of the value indicators (e.g., for the 10 value dimensions, see Schmidt et al., 2021) to reduce their impact, because reliability was low, factor analytic fit in the German sample of the ESS was insufficient, and because these procedures did not result in a considerably lower relative impact of the values on the milieu solution.

#### Social cohesion across social milieus

To investigate differences in the “trust” and “concerned compliance” factor scores across social milieus we use the “Bakk-Kuha” method (Bakk and Kuha, 2018, 2021). This method accounts for measurement error in the latent milieu variable in two steps: First, an LCA is conducted as described in section “Typology of social milieus.” Second, a structural model adding outcomes is calculated. Here, the parameters of the measurement model obtained in the first step are fixed so that the milieu estimation stays the same. The Bakk-Kuha method is especially helpful when the sample sizes between the LCA and the structural model differ, as in our case. In Latent GOLD^®^ 6.0, a user-friendly version of the two-step method has been implemented that saves individuals' milieu-specific probability densities in the first step for their use in the second step (Vermunt and Magidson, 2021). We estimate (a) a two-step model that regresses the milieus on the cohesion factors as outcomes and (b) a model that additionally includes the effect of sociodemographic covariates (sex, age, and region) on the cohesion factors (see the Latent GOLD^®^ 6.0 syntax in the Supplementary material).

##  Results

### A Latent Class Analysis of social milieus

The LCA, described in section “Typology of social milieus”, provides three types of output: (1) the *sizes or percentage shares of the social milieus* and (2) *milieu-specific estimates of the indicators:* (a) estimated proportions of education and income as categorical indicators and (b) means of the 21 person-centered value items. (3) Additionally, *coefficients of covariates and outcomes* can be estimated using the Bakk-Kuha method (see section Social cohesion across social milieus). The nine sociodemographic milieus can be described based on these outputs. In addition to the milieu indicators we report socio-demographic information on age, sex, and region—which do not affect milieu composition (see Table 1, where the value items are condensed into the four higher-order value dimensions, and Supplementary Table S4 including all 21 value items).

**Table 1:** A model of social milieus: Latent Class Analysis of socioeconomic position and basic human values.

**Milieus**	**1**	**2**	**3**	**4**	**5**	**6**	**7**	**8**	**9**	**Overall**
Size (in %)	17.0	7.2	7.8	9.9	4.2	10.4	8.4	16.6	18.6	100.0
Size (case numbers)	435	192	190	230	110	261	219	332	501	2,470
**Socioeconomic dimension**										
**Equalized household income, quintile groups (in %)**										
1	7.1	13.1	10.6	17.5	14.3	23.2	27.0	27.3	27.3	19.6
2	12.9	18.7	16.6	21.9	19.6	25.0	26.6	26.7	26.7	22.0
3	17.7	20.1	19.5	20.6	20.3	20.3	19.7	19.7	19.7	19.6
4	22.7	20.3	21.4	18.2	19.8	15.5	13.7	13.6	13.6	17.2
5	39.6	27.7	31.9	21.9	26.0	15.9	12.9	12.7	12.7	21.7
**Highest educational degree (in %)**
Low	7.6	11.1	20.5	25.2	39.6	40.8	40.5	44.5	57.7	33.6
Intermed.	26.9	30.7	36.0	37.2	36.9	36.6	36.7	35.8	30.9	33.4
High	65.5	58.2	43.5	37.6	23.5	22.6	22.8	19.7	11.4	33.1
Cultural dimension: Higher-order values^*^										
Openness	−0.26	0.72	0.67	−0.3	0.05	0.55	−1	−0	−0.3	−0.04
Conservation	−0.14	−1.1	−1	−0	0.2	−0.4	0.71	0.01	0.55	−0.05
Self-transcendence	0.85	1.34	0.52	1.44	0.02	0.93	1.07	0.33	0.83	0.82
Self-enhancement	−0.47	−1.1	−0.2	−1.4	−0.4	−1.4	−1	−0.4	−1.4	−0.88
**Sociodemographic characteristics**										
Sex: Women (in %)	50.2	49.0	34.7	64.3	39.7	62.4	61.2	32.9	58.4	50.7
Age (in years)	44.7	41.9	31.5	53.2	55.9	45.9	54.2	43.1	63.3	48.9
Region: East Germany (in %)	14.1	9.8	12.0	8.0	14.6	20.5	25.2	16.9	27.0	17.5

Source: ESS8, 2016, n = 2,470, own calculations.

^*^The averages of the 21 person-centered value items are aggregated to the four higher-order value dimensions by calculating means.

**Figure 3:**
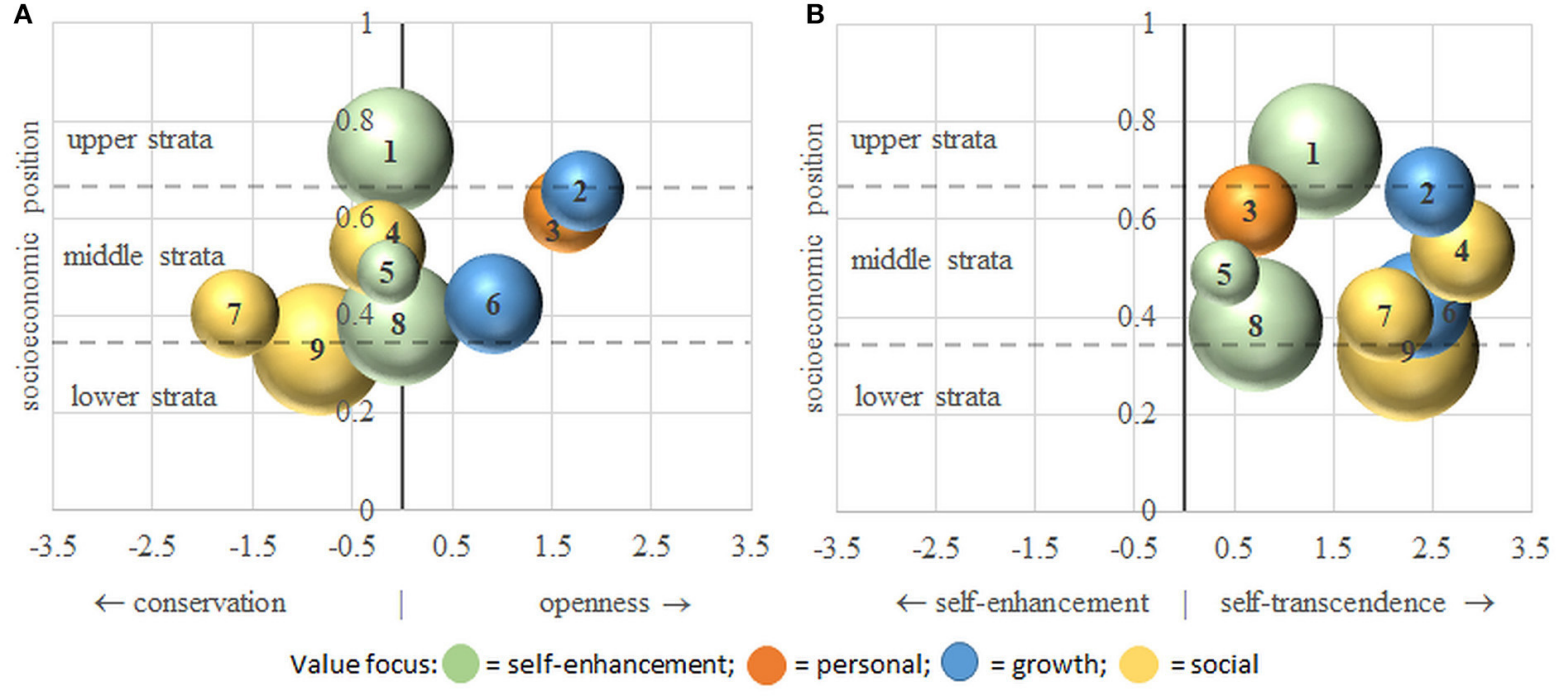
A new model of social milieus: Latent Class Analysis of socioeconomic position and basic human values. Source: ESS8, 2016, *n* = 2,470, own calculations. The social milieus' socioeconomic positions are plotted against the value axis from conservation to openness **(panel A)**, and the value axis from self-enhancement to self-transcendence **(panel B)**. Milieus with similar value foci are assigned the same color.

For the purpose of presentation, similar to Magun et al. (2016), we plot the milieus' socioeconomic positions (*y*-axes) against their positions on each of the two value dimensions (*x*-axes) in two bubble charts (see Figure 3). The sizes of the bubbles correspond to the sizes of the social milieus. For presenting the milieus' socioeconomic position, income and education are treated as continuous variables so that the milieu-specific means can be calculated, transformed onto a common scale with a minimum of 0 and a maximum of 1, and then averaged. A value of “1” (“0”) indicates the highest (lowest) average score of the milieu members, that is the 5^th^ (1.) income quintile and upper (lower) secondary school. The status axis is additionally divided into three strata corresponding to the lower, middle, and upper third of the analytically possible range. The social milieus' value positions are presented on two axes, one ranging from conservation to openness and the other from self-transcendence to self-enhancement. To identify the milieus' positions on these axes, the milieus' averages of the 21 person-centered value items are first aggregated to the four higher-order value dimensions by calculating means. These dimensions are then further condensed into the two value axes by subtracting (1) conservation from openness and (2) self-enhancement from self-transcendence. For better interpretation, each milieu is assigned a color indicating its value focus, i.e., its position on both value axes relative to the other milieus. For example, we assign a personal value focus to a milieu that endorses openness values (panel A of Figure 3) and self-enhancement values (panel B of Figure 3) more strongly than other milieus. If a milieu holds average values on one value dimension, we name its focus after the higher-order value it tends to on the other value dimension (e.g., self-enhancement focus).

At this point, we refrain from giving concrete names to each milieu. This procedure requires comprehensive analyses in terms of criterion validity, i.e., systematic milieu differences in sociodemographic characteristics, attitudes, and practices. This is in line with other milieu approaches, notably the Sinus^®^ milieus, for which naming is the result of a process of extensive research (Flaig et al., 1994). Instead, we number the milieus according to their socioeconomic status, classify them into lower, middle, and upper socioeconomic strata, and finally color and designate them according to their value foci.

Figure 3 shows that, overall, considerable heterogeneity concerning milieu differentiation along the stratification and value axes can be observed. The milieus are clearly stratified by socioeconomic position (income and education). Although the boundaries are somewhat arbitrary, roughly, one upper-class milieu (1), two upper-middle-class milieus (2, 3), two middle-class milieus (4, 5), three lower-middle-class milieus (6, 7, 8), and one lower-class milieu (9) can be identified^8^.

Social milieus are also differentiated according to their positions on the two value axes. In every socioeconomic stratum milieus with different value profiles are observed. In line with the literature, there is an overall tendency for milieus in higher socioeconomic positions (compared to lower positions) to endorse openness values more and conservation values less strongly. The value axis from self-transcendence to self-enhancement, in turn, is relatively independent of the socioeconomic position. Furthermore, all milieus tend more toward self-transcendence than self-enhancement, but there are considerable differences in the extent of this tendency.

Milieus 1, 5, and 8 (green) from the upper-, middle-, and lower-middle-class hold relatively high self-enhancement values and average values on the axis from conservation to openness. While there is no milieu with a clear protection focus, milieu 5 (size: 4%) resembles Reckwitz (2019) “old” middle class most as it shows relatively high incomes and intermediate education. However, this milieu is much smaller than presumed by Reckwitz. It is also smaller than the Sinus^®^ milieu of the bourgeois or nostalgic middle class (11%) which has been identified as the core milieu of the old middle class. Furthermore, Milieu 5 is similar in its socioeconomic and value profile to the achievement-oriented milieu (El-Menouar, 2021). The milieus 1 and 8, in turn, are not captured by previous milieu typologies. Milieu 1 (17%) is less conservative than the conservative upscale Sinus^®^ milieu and much older than the achievement-oriented milieu of El-Menouar (2021). Milieu 8 (17%) is located somewhere between the lower ranks of the nostalgic and adaptive-pragmatic middle class of the Sinus^®^ typology, which are classified as part of the old middle class by Reckwitz (2019). However, the low average of socioeconomic positions marks it as a separate milieu.

The upper-middle-class milieu 3 (8%) holds a person focus as openness and self-enhancement values are endorsed (orange). The milieu only weakly resembles the performer milieu of the Sinus^®^ typology or the individualistic materialist milieu of El-Menouar (2021) typology as its members are much younger on average.

Milieus 2 and 6 from the upper-middle and lower-middle class (blue) hold a growth value focus (high self-transcendence and openness). This focus is stronger in milieu 2 (7%) which resembles the expeditive Sinus^®^ milieu as part of Reckwitz (2019) “new” middle class. Milieu 6 (10%) endorses strong hedonism values and some aspects of tradition and security values. In this respect, milieu 6 resembles both the adaptive-pragmatic middle class of the Sinus^®^ milieus and the humble humanists of El-Menouar (2021) typology. Its lower socioeconomic position (especially in education) disqualifies it as a “new” middle-class milieu.

Finally, the milieus 4, 7, and 9 hold a social value focus (yellow), albeit with varying positions on the two value axes. All of these milieus have a large proportion of older or female members. Middle-class milieu 4 (10%) is the least conservative of these three milieus, self-transcendence values are predominant. Insofar as its relatively central position on the conservation-vs.-openness axis is due to high modesty and humbleness as well as low conformity and hedonism, this milieu resembles El-Menouar (2021) humble humanists. Regarding its values, milieu 4 thus resembles Reckwitz (2019) “new” middle class, but due to its only average education, it is not considered as such. The lower-middle-class milieu 7 (8%) is the most conservative. The lower-class milieu 9 (19%) lies in-between milieus 4 and 7 on the conservation-vs.-openness axis. The characterization of milieus 7 and 9 as traditional (Sinus^®^ Institute., 2020; Beckmann and Schönauer, 2021) or safety-oriented conservatives (El-Menouar, 2021) fails to recognize the high endorsement of self-transcendence values.

Within the lower classes, our milieu typology could neither detect a precarious milieu with a rather average value focus (Sinus^®^) nor a hedonistic (Sinus^®^) nor alternative milieu (Beckmann and Schönauer, 2021) with low socioeconomic positions and high openness values. If any milieu has an average value focus, it is the upper-class milieu 1, and openness values are stronger in the upper-middle-classes. Apart from these exceptions, the social milieus we expected to exist in our general expectations deduced from the literature (see section “A new model of social milieus”) emerged in our analyses.

### Milieu differences in social cohesion

Having described our milieu typology, we now turn to the investigation of milieu differences in “trust” and “concerned compliance.” We regressed the “trust” and “concerned compliance” factors on the nine milieus using the Bakk-Kuha method described in section Social cohesion across social milieus. Additionally, we ran a model that also controls for the effect of age, sex, and region (East Germany) on the cohesion factors. These covariates decrease the sizes of the milieu coefficients, but only to a small degree, and do not change their direction or significance (the results of this analysis are presented in Supplementary Table S5). Here, we focus on the model without covariates as we are primarily interested in overall milieus differences.

At first, a look at the single items comprising the two cohesion factors reveals an only intermediate level of “trust” in social cohesion regarding item-specific approval rates (“agree”/“strongly agree”) which range from 54 to 63%. These rates are much higher for the concerned compliance factor (68% to 96%). This finding is not in line with the thesis of a rally effect that postulates strong homogeneity and strong overall social cohesion.

Bivariate correlations between the milieu components and the social cohesion factors show that higher trust is weakly associated with a higher socioeconomic position and higher self-transcendence values (Table 2). Concerned compliance is positively associated with conservation and self-transcendence and negatively associated with openness and self-enhancement, and tends to be negatively associated with education. This is in line with earlier findings. However, these correlations only inform about general associations between variables. They do not reveal heterogeneity between social groups, i.e., they neither inform about group size, nor which group takes which position in the social space comprised of the socioeconomic and cultural dimensions, nor show the strength of the opposition between groups.

**Table 2:** Bivariate correlations between the milieu components and the social cohesion factors.

	**Trust**	**Compliance**	**Income**	**Education**	**Openness**	**Conservation**	**Self-Transcendence**
Compliance	0.373^***^						
Income	0.101^***^	0.017					
Education	0.052	−0.077^***^	0.319^***^				
Openness	−0.050	−0.129^***^	0.050^***^	0.044^***^			
Conservation	0.037	0.138^***^	−0.139^***^	−0.220^***^	−0.731^***^		
Self-Transc.	0.086^***^	0.150	0.038^***^	0.127^***^	−0.164^***^	−0.150^***^	
Self-Enhanc.	−0.060	−0.150	0.095^***^	0.134^***^	−0.086^***^	−0.337^***^	−0.482^***^

Source: RISC pilot study (2020), matched with the ESS8 (2016), n = 526, own calculations.

The 21 value items are condensed to the four higher-order value dimensions for the ease of interpretation.

^***^p ≤ 0.1.

**Figure 4:**
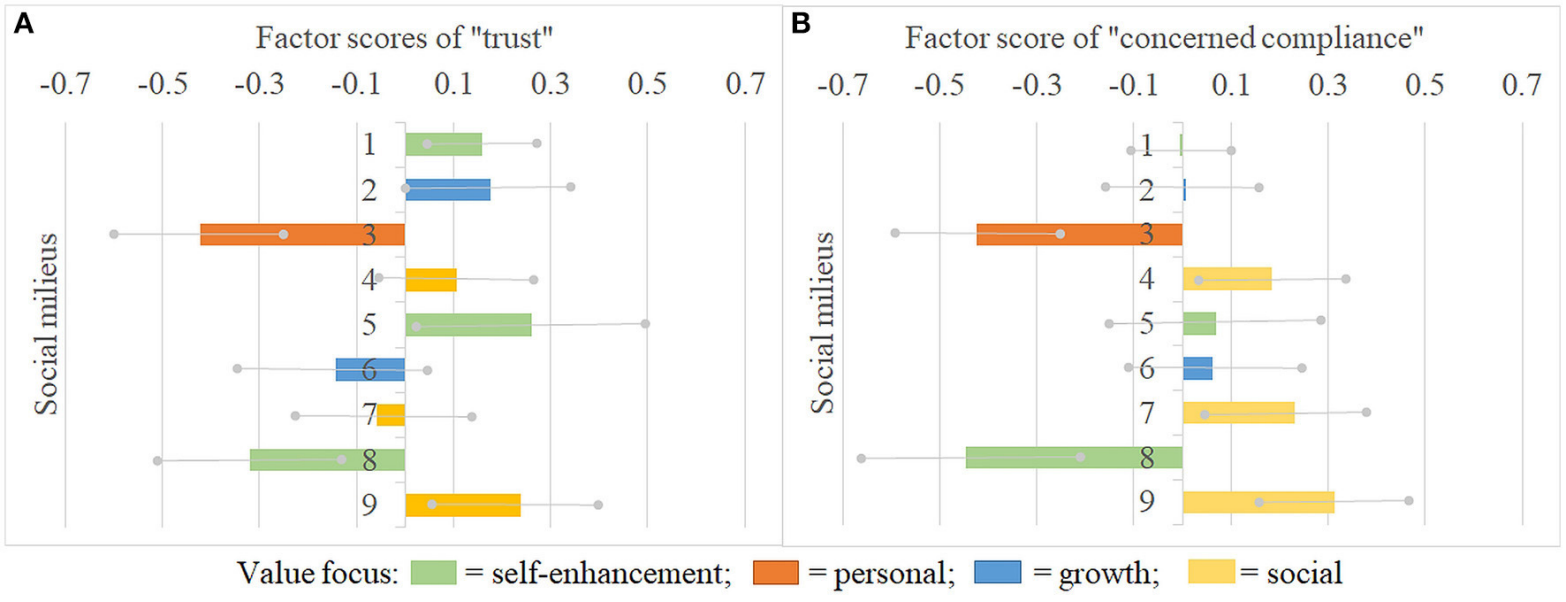
Factor scores of “trust” **(panel A)** and “concerned compliance” **(panel B)** by social milieus. Source: RISC pilot study 2020, merged with ESS8, 2016, *n* = 526, own calculations. Note: The milieu-specific factor scores represent deviations from the mean factor score. Gray lines indicate 90% confidence intervals (We think, these rather broad intervals are justified because of the small case numbers per milieu.). Milieus are numbered by level of socioeconomic status and colored by value focus (see Figure 3).

Hence, we use the milieu model to analyze group differences in the social cohesion factors, thereby going beyond what can be shown by variable-based analysis. Considerable heterogeneity between social milieus regarding both cohesion factors can be observed. Figure 4 presents a bar chart of the endorsement of “trust” (panel A) and “concerned compliance” (panel B) across social milieus. The milieu-specific factor scores can be interpreted as deviations from the overall mean which is zero. The milieus are again numbered by their level of socioeconomic status and colored by their value foci.

In accordance with our expectations, and not surprisingly given the bivariate correlations, milieus with a social value focus (milieus 4, 7, and 9) show high concerned compliance, and two milieus that hold self-enhancement (milieu 8) and personal values (milieu 3) show low concerned compliance. Turning to the socioeconomic position, it is noticeable that despite the positive correlation between trust and socioeconomic position, a milieu with one of the highest levels of trust (milieu 9) is to be found in the lower class, and the milieu with the lowest trust (milieu 3) in the upper-middle-class. Furthermore, it can be seen that concerned compliance tended to be closer to the average among the higher socioeconomic positions with milieu 3 as a great exception.

Considering the different modes of social cohesion in terms of constellations of trust and concerned compliance, one central finding stands out. The upper-middle class milieu 3 and the lower-middle-class milieu 8 have a similar mode of social cohesion with low trust and low concerned compliance. While milieu 3 stands out as the only milieu with a personal value focus, milieu 8 holds self-enhancement values but is located in the middle of the openness vs. conservation axis. In contrast to these milieus, the lower-class social value milieu 9 exhibits a diametrical mode of cohesion with high trust and high concerned compliance.

Similar value foci do not always bring about similar modes of social cohesion across all socioeconomic positions. For example, the upper-middle-class milieu 2 and the lower-middle-class milieu 6 both have a growth focus, but the latter has a lower socioeconomic position as well as lower levels of trust. Milieu 6 thereby rather resembles the indifferent adaptive-pragmatic milieu (Sinus^®^ Institute., 2020) than the trusting humble humanists (El-Menouar, 2021). Milieu 2, in turn, resembles upper-class milieu 1 in showing average levels of concerned compliance despite the different value focus. Possibly, these milieus are less concerned about the pandemic due to their high socioeconomic position. It is furthermore noticeable that the lower-middle-class milieu 8 on the one hand and milieus 1 and 5 on the other hand differ greatly in their attitudes toward cohesion, especially concerning trust—although all of these milieus have a self-enhancement value focus. The higher social standing and relative economic security might lead the latter two milieus to trust in social cohesion. These results show that the specific combinations of socioeconomic positions and value profiles are highly relevant for milieus' modes of social cohesion.

Regarding Reckwitz (2019) distinction between the ‘old' middle class (milieu 5 in our model) and the “new” middle class (milieu 2 in our model), both classes show relatively high trust and average levels of concerned compliance. Thus, they resemble each other in their modes of cohesion and are not central conflicting social groups as presumed by Reckwitz—at least concerning social cohesion. Instead, the small upper-class milieu 3 with a person focus and the large lower-middle-class milieu 8 with a self-enhancement focus (adding up to 25%) are on the lower extreme ends of both cohesion factors. They oppose milieus with a social focus (milieu 4, 7, and 9; adding up to 47%) on matters of concerned compliance, and they confront milieus with a rather high socioeconomic position (especially milieus 1, 2, 5; adding up to 28%) and milieu 9 (19%) in their trust in social cohesion.

## Discussion

The aim of this paper was to uncover heterogeneity and potential conflicts within the German population about social cohesion during the COVID-19 pandemic by analyzing large subgroups within the society. The concept of social milieus—similar to “cultural class analysis” but without the ambiguity of the class term—lends itself to such a subgroup analysis. It addresses the interrelation of socioeconomic stratification and cultural aspects in constituting large latent social groups. The concept has been introduced particularly for the analysis of social cohesion as a group-specific form of social integration. We assume that social milieus develop specific modes of social cohesion and that different modes express conflicting viewpoints which are the base of potential social conflicts. The concept of social milieus is thus particularly suited to analyze the social integration of conflicting groups on the societal level during a crisis like the COVID-19 pandemic.

Extant milieu approaches, however, suffer from theoretical and empirical deficiencies. El-Menouar (2021) typology misses a stratification dimension, and it is unclear how this dimension differentiates the Sinus^®^ Institute. (2020) typology. Furthermore, both the Sinus^®^ and Beckmann and Schönauer (2021) milieu typologies are composed of a one-dimensional value axis. To overcome these limitations, we use a new model of social milieus. Milieus are constituted by a socioeconomic dimension, composed of education and household income, and a cultural dimension, operationalized through the multi-dimensional approach of Schwartz (1992) basic human values. This model differs from the previous approaches in three ways: first, it directly considers socioeconomic stratification in the milieu composition. Second, values are captured comprehensively and in their potentially conflictual relation toward each other. Importantly, in addition to the conservative vs. openness axis, an axis ranging from self-enhancement to self-transcendence allows for a finer breakdown of value constellations. Third, the typology can be readily operationalized and replicated with publicly available large-scale survey data. We use this milieu typology to empirically investigate expectations concerning milieu-specific modes of social cohesion during the COVID-19 pandemic, derived from previous milieu analyses. Trust in social cohesion and concerned compliance with measures, reflecting trust and conformity as ingredients of social cohesion, are analyzed. We use the European Social Survey (ESS) Round 8 (2016) for the identification of social milieus and the RISC pilot study (2020), which can be merged with the ESS data, for the analysis of social cohesion.

Our analyses reveal more heterogeneity in the first wave of the COVID-19 pandemic and its aftermath than the “rally-round-the-flag” effect presumes. The findings on milieu differences support some expectations we have formulated based on previous literature but also provide new insights that could not be captured by extant milieu typologies. As expected, a milieu with higher socioeconomic status and a personal value focus was identified that deviates from the “rally-around-the-flag” response by showing particularly low levels of trust and compliance. A similar mode of social cohesion prevails in a lower-middle-class milieu with a self-enhancement value focus. This rather large milieu could not be detected by previous typologies due to missing dimensions in the operationalization. As expected, especially compliance, and to a lower extent trust, is high in milieus with a social value focus, no matter what their socioeconomic position is. In contrast to previous studies, however, trust and compliance are exceptionally strong in the lower-class social value milieu. Thus, the finding of the Sinus^®^ Institute. (2020) typology of a distrustful and non-compliant precarious social milieu should be differentiated: Within the lower socioeconomic ranks, two social milieus with different modes of social cohesion due to different compositions in the value dimension can be identified. Hence, the highest potential for conflict with respect to modes of social cohesion can be observed between the social value-focused lower-class milieu and the self-enhancement and personal value-focused lower- and upper-middle-class milieus. This potential conflict seems to be more about basic human values than socioeconomic resources. Beyond this general conflict line, non-negligible heterogeneity in modes of cohesion and associations with milieu-defining characteristics exists. For example, we clearly identified a “new” middle class milieu (Reckwitz, 2019) with a high socioeconomic position and a growth value focus, showing above-average levels of trust and average levels of compliance. However, no particular conflict between the “new” and “old” middle classes (Reckwitz, 2019) could be observed concerning trust and compliance.

Our research is not without limitations. Regarding the empirical analysis, first, due to data limitations, we only address two ingredients of social integration: trust and conformity. Future research looking into all four ingredients might be able to detect a wider variety of modes of social cohesion. Second, the small sample size of the RISC pilot study restricts generalizability and the potential to detect milieu differences. Third, the operationalization of the milieu concept presented here is the first step toward a full account of our theoretical model. Hence, future research might further improve the milieu typology. Especially, sub-milieus below the general milieus presented here may be analyzed as is milieu segmentation due to sociodemographic characteristics. For example, investigating age differences might better approximate individual lifeworlds and specific modes of social cohesion. Fourth, the current typology has to be further validated. For example, cross-country comparisons would allow us to go beyond country-specific peculiarities. Finally, the quantitative milieu analyses should be complemented with qualitative data to bring subjective meaning into milieu analysis. We already made use of partial information from the qualitative RISC panel, but this perspective has to be developed systematically.

At the same time, the present research overcomes several current limitations. First, we use a milieu typology for our analyses that is replicable with large-scale survey data and appropriately considers socioeconomic stratification and multidimensional cultural values. Second, building on theoretical considerations connecting social cohesion and social milieus, we were able to empirically discover milieu differences in the endorsement of two ingredients of social integration in the context of the COVID-19 pandemic, reflecting milieu-specific modes of social cohesion. The RISC pilot study allows us to assess the specific situation during the first wave of the pandemic and its aftermath. A future analysis of the ESS10 (2020) might be worthwhile as it includes a module on cohesion during the COVID-19 pandemic (Hanson et al., 2021). Yet, the module is restricted to institutional trust and does not directly assess the acceptance of restrictions. Moreover, our previous analyses can later be continued with the first wave of the RISC panel conducted in 2021. The extension of the analyses particularly allows for the inclusion of later waves of the pandemic as well as longitudinal analyses—but it does not capture the early phase of the pandemic. In sum, our milieu approach enriches current debates about social integration and cohesion during the COVID-19 pandemic by providing a group perspective on which later analyses can build.

## Data availability statement

Publicly available datasets were analyzed in this study. This data can be found at: https://ess-search.nsd.no/.

## Ethics statement

Ethical review and approval was not required for the study on human participants in accordance with the local legislation and institutional requirements. The patients/participants provided their written informed consent to participate in this study.

## Author contributions

AS wrote chapter 4 and performed the Latent Class Analysis of the final model and implemented the Bakk-Kuha method for associating cohesion factors and social milieus. TS wrote the first draft of chapters 1, 2, 3, 5, and 6 and performed the final analysis, including the factor analysis and the Bakk-Kuha method. AS and TS conducted several robustness checks and sensitivity analyses. All authors contributed to conception and design of the study, manuscript revision, read, and approved the submitted version.

## Footnotes

^1^The development of these indicators is documented by the WSI (2020), Busemeyer et al. (2021a,b), the “Mannheim Corona Study” (https://www.uni-mannheim.de/gip/corona-studie/) and, on a weekly basis, the COSMO–COVID-19 Snapshot Monitoring (https://projekte.uni-erfurt.de/cosmo2020/web/).

^2^Technically, when speaking of social cohesion at the societal level, the term “societal cohesion”—similar to the German term “gesellschaftlicher Zusammenhalt”—would be more appropriate. We nevertheless use “social cohesion” as it is implemented in the literature.

^3^Unfortunately, the French term “milieu” is mostly translated as “environment” (e.g., Durkheim, 1897[2002], p. 135ff) and thus loses part of its meaning, e.g., as a medium relating social actors. In German, the word “Milieu” is almost always used in a sociological or historical context, while “Umwelt” more generally designates the environment.

^4^We use the identical procedure as in Groh-Samberg (forthcoming).

^5^Technically, we conduct a mix of LCA (for the categorical socioeconomic indicators) and Latent Profile Analysis (for the person-centered and thus quasi-metric value indicators).

^6^The Vuong-Lo-Mendell-Rubin adjusted likelihood ratio test (VLMR-LRT), which compares the fit improvement between two adjacent class solutions, was not further consulted because it did not get insignificant for any considered class solution.

^7^The results of these additional analyses can be provided on request.

^8^It is important to note that we speak of an “upper class” only in terms of education and income. A “proper” upper class, who makes a living solely from capital assets, cannot be identified and is underrepresented in our sample (see Reckwitz, 2019).
