# Supplementary material for: Trust and compliance: Milieu-specific differences in social cohesion during the COVID-19 pandemic in Germany
Source: Front Sociol. 2022 Dec 21;7:989831. doi: 10.3389/fsoc.2022.989831 (PMC9811820; doi:10.3389/fsoc.2022.989831)
Supplement: Supplementary file 1 [file Data_Sheet_1.PDF]

Trust and compliance: Milieu-specific differences in social cohesion during the Covid-19 pandemic in Germany

Schröder, Tim; Speer, Anne; Sachweh, Patrick and Groh-Samberg, Olaf

In: Frontiers in Sociology. Research Topic: The COVID-19 Pandemic and Social Cohesion across the Globe, edited by Mandi M. Larsen, Georgi Dragolov and Jan Delhey

Supplementary Material

Table S1. ESS 8 (2016) and RISC pilot study (2020) by socio-structural characteristics

		ESS 8, n=2852	RISC study*, matched with ESS full sample, n=589	RISC study*, analysis data set, n=526
		in %, weighted	in %, weighted	in %, unweighted
Gender	male	49.1	48.9	50.6
	female	50.9	51.1	49.4
Age in years	15-18	5.1	6.5	6.1
	18-34	22	24.6	22.5
	35-49	22.6	20.5	23.5
	50-64	28.1	29.9	30.6
	65-	22.2	18.5	17.4
Region	West Germany	82.8	79.6	65.6
	East Germany	17.2	20.4	34.4
Highest educational degree	Low	33.5	24.6	16.9
	Intermediate	33.1	30.7	32.7
	High	33.3	44.9	50.4
Equalized household income	1. quintile	20.4	15.1	12.7
	2. quintile	21.8	19.6	19.1
	3. quintile	19.1	19.2	20.3
	4. quintile	17.1	20.5	21.7
	5. quintile	21.6	25.6	26.2

* All variables measured in ESS8, 2016

Table S2. Cohesion factors (rotated, oblique, quartimin method in Stata® 15)

	Scale Reliability Coefficient	Eigen-values	Factor loadings	
			Trust in cohesion	Concerned compliance
Trust in cohesion	0.70	1.59		
The handling of the coronavirus shows that we can rely on social cohesion (Coro1_6)			0.689	-0.019
The handling of the coronavirus shows the bad condition of social cohesion (Coro1_7)			-0.626	-0.014
I trust that my fellow citizens accept measures to contribute to contain the virus (Coro1_5)			0.615	-0.071
Concerned compliance	0.60	1.24		
I accept the restrictions in order to contribute my share to contain the virus (Coro1_3)			0.040	0.562
I think, the measures to contain the coronavirus are excessive (Coro1_4)			-0.053	-0.642
I am concerned about the spreading of the coronavirus (Coro1_1)			-0.128	0.516
Item with cross-loadings				
I trust that necessary measures are taken to contain the coronavirus (Coro1_2)			0.466	0.301

Source: RISC 2020, n=565, own calculations

Latent Gold 6.0 syntax of Latent Class Analysis

Step 1: Estimate 9-class LCA measurement model, safe class-specific log densities for step 2

```
options
   maxthreads=all;
   algorithm
      tolerance=1e-008 emtolerance=0,01 emiterations=250 nriterations=50 ;
   startvalues
      seed=0 sets=400 tolerance=1e-005 iterations=200;
   bayes
      categorical=1 variances=1 latent=1 poisson=1;
   montecarlo
      seed=0 sets=0 replicates=500 tolerance=1e-008 allchi2 lldiff alpha=0,05;
   quadrature  nodes=10;
   missing  excludeall;
   output
      parameters=effect  betaopts=wl standarderrors=robust profile probmeans=posterior
      loadings bivariateresiduals estimatedvalues=model reorderclasses iterationdetail;
   outfile
      'lca9cl.sav' logdensity keep=sex age agea eastwest covtrust covcompl;
variables
   caseid
      idno;
   caseweight
      pspwght;
   dependent inc ordinal, educ ordinal scores=(  1  2  3) , peqopt continuous , pudrst continuous ,
      penv continuous, phlppl continuous , plylfr continuous , pmodst continuous ,
      ptrad continuous , pfrule continuous , pbhprp continuous , pstrgv continuous ,
      psafe continuous , prspot continuous, prich continuous , pshabt continuous ,
      psuces continuous , pgdtim continuous , pfun continuous , pdiff continuous ,
      padvnt continuous , pcrtiv continuous , pfree continuous ;
   latent
      milieus nominal 9;
equations
   milieus <- 1;
   inc educ  <- 1 + milieus;
   peqopt-pfree <- 1 + milieus;
```

Step 2 (Model 1+3 in Table S5): Estimate structural model with latent independent variable (log densities of nine milieus) and trust and concerned compliance as (distal) outcomes

```
options
   maxthreads=all;
   algorithm
      tolerance=1e-008 emtolerance=0,01 emiterations=250 nriterations=50 ;
   startvalues
      seed=0 sets=400 tolerance=1e-005 iterations=200;
   bayes
      categorical=1 variances=1 latent=1 poisson=1;
   montecarlo
      seed=0 sets=0 replicates=500 tolerance=1e-008;
   quadrature  nodes=10;
   missing  excludeall;
   output
      parameters=effect  betaopts=wl standarderrors=robust profile probmeans=posterior
      loadings bivariateresiduals predictionstatistics=conditional
      estimatedvalues=model reorderclasses iterationdetail;
variables
   dependent covtrust continuous, covcompl continuous;
```

```
            latent
        milieus nominal logdensity=(logdensity1 logdensity2 logdensity3 logdensity4
          logdensity5 logdensity6 logdensity7 logdensity8 logdensity9);
equations
   covtrust <- 1 + milieus;
   covcompl <- 1 + milieus;
   covtrust;
   covcompl;
```

Step 2 (Model 2+4 in Table S5): Estimate structural model with latent independent variable (log densities of nine milieus) plus sex, age, and region (East Germany) as covariates directly effecting trust and concerned compliance as (distal) outcomes

```
options
   maxthreads=all;
   algorithm
      tolerance=1e-008 emtolerance=0,01 emiterations=250 nriterations=50 ;
   startvalues
      seed=0 sets=400 tolerance=1e-005 iterations=200;
   bayes
      categorical=1 variances=1 latent=1 poisson=1;
   montecarlo
      seed=0 sets=0 replicates=500 tolerance=1e-008;
   quadrature  nodes=10;
   missing  excludeall;
   output
      parameters=effect  betaopts=wl standarderrors=robust profile probmeans=posterior
      loadings bivariateresiduals predictionstatistics=conditional
      estimatedvalues=model reorderclasses iterationdetail;
variables
   dependent covtrust continuous, covcompl continuous;
   independent sex nominal , agea ,  eastwest nominal;
   latent
      milieus nominal logdensity=(logdensity1 logdensity2 logdensity3 logdensity4
         logdensity5 logdensity6 logdensity7 logdensity8 logdensity9);
equations
   covtrust <- 1 + milieus + sex + agea + eastwest;
   covcompl <- 1 + milieus + sex + agea + eastwest;
   covtrust;
   covcompl;
```

Table S3. Latent Class Analysis, model summary

No. of milieus	LL	BIC(LL)	AIC(LL)	AIC3(LL)	CAIC(LL)	SABIC(LL)	No. para-meters	p-value VLMR-Test	Entropy R^2
1	-83410.772	167195.979	166917.544	166965.544	167243.979	167043.471	48		1.000
2	-81990.520	164542.692	164125.041	164197.041	164614.692	164313.931	72	0.000	0.743
3	-81157.158	163063.184	162506.316	162602.316	163159.184	162758.170	96	0.000	0.749
4	-80835.963	162608.011	161911.926	162031.926	162728.011	162226.743	120	0.000	0.725
5	-80567.289	162257.882	161422.579	161566.579	162401.882	161800.360	144	0.000	0.736
6	-80305.086	161920.693	160946.172	161114.172	162088.693	161386.917	168	0.000	0.742
7	-80128.136	161754.009	160640.272	160832.272	161946.009	161143.980	192	0.000	0.736
8	-79960.331	161605.616	160352.661	160568.661	161821.616	160919.333	216	0.000	0.738
9	-79791.866	161455.904	160063.733	160303.733	161695.904	160693.368	240	0.000	0.751
10	-79651.322	161362.032	159830.644	160094.644	161626.032	160523.242	264	0.000	0.760
11	-79519.648	161285.903	159615.297	159903.297	161573.903	160370.859	288	0.000	0.764
12	-79390.659	161215.141	159405.318	159717.318	161527.141	160223.843	312	0.000	0.769
13	-79274.789	161170.619	159221.578	159557.578	161506.619	160103.067	336	0.000	0.772
14	-79179.497	161167.252	159078.995	159438.995	161527.252	160023.447	360	0.001	0.775
15	-79089.886	161175.246	158947.772	159331.772	161559.246	159955.188	384	0.014	0.777

Source: ESS8, 2016, n=2470, own calculations

Note: LL=Log-Likelihood; BIC=Bayesian Information Criterion; AIC=Akaike Information Criterion; AIC3=Akaike Information Criterion 3; CAIC=Consistent Akaike Information Criterion; SABIC=sample size adjusted BIC; VLMR-Test=Vuong-Lo-Mendel-Rubin Test

Figure S1. Latent Class Analysis, 13-class solution

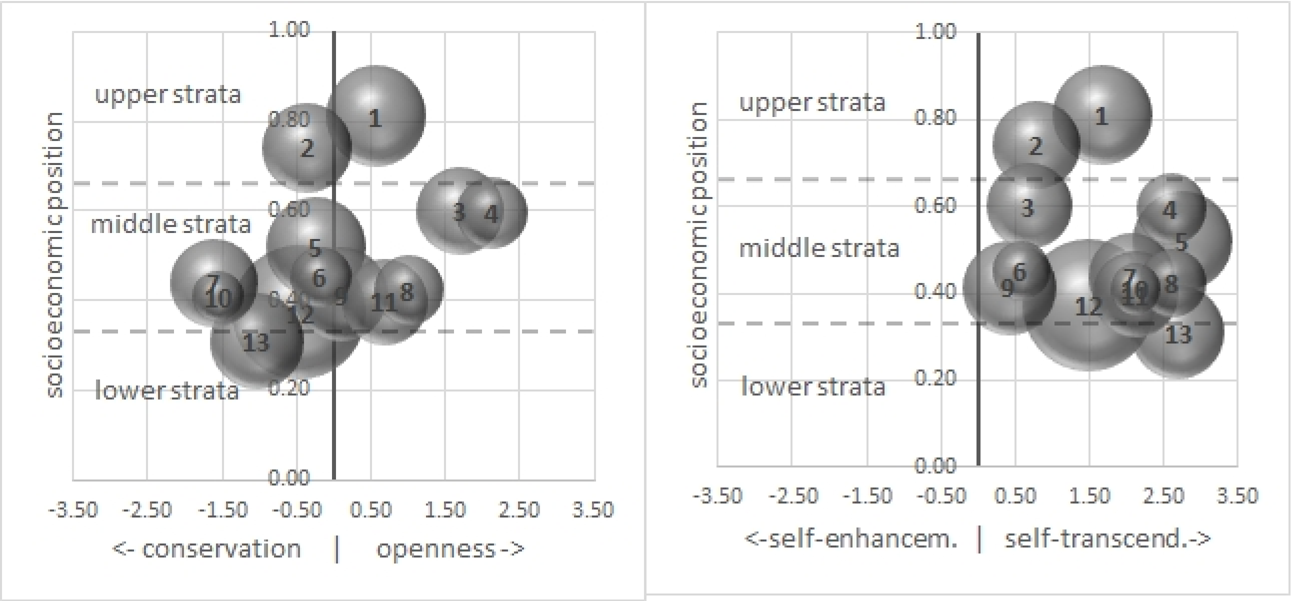

Source: ESS 8, 2016, n=2470, own calculations

Table S4. LCA results, 9-class solution; estimated values of milieu indicators

Milieus		1	2	3	4	5	6	7	8	9	Overall
Size (in %)		17	7.2	7.8	9.9	4.2	10.4	8.4	16.6	18.6	100
Size (case numbers)		435	192	190	230	110	261	219	332	501	2,470
Equalized household income quintile groups (in %)	1. quintile	7.1	13.1	10.6	17.5	14.3	23.2	27.0	27.3	27.3	19.6
	2. quintile	12.9	18.7	16.6	21.9	19.6	25.0	26.6	26.7	26.7	22.0
	3. quintile	17.7	20.1	19.5	20.6	20.3	20.3	19.7	19.7	19.7	19.6
	4. quintile	22.7	20.3	21.4	18.2	19.8	15.5	13.7	13.6	13.6	17.2
	5. quintile	39.6	27.7	31.9	21.9	26.0	15.9	12.9	12.7	12.7	21.7
Highest educational degree (in %)	low	7.6	11.1	20.5	25.2	39.6	40.8	40.5	44.5	57.7	33.6
	intermediate.	26.9	30.7	36.0	37.2	36.9	36.6	36.7	35.8	30.9	33.4
	high	65.5	58.2	43.5	37.6	23.5	22.6	22.8	19.7	11.4	33.1
Socioeconomic position (status)[a]		0.74	0.66	0.62	0.54	0.49	0.42	0.4	0.39	0.33	0.5
Human Values Scale (portrait values questionnaire, PVQ-21).[b] *Means of person-centered value items across milieus. Person-centering implies subtracting a persons' value of an item (ranging from 1 "not like me at all" to 5 "very much like me") from the persons' mean of all 21 items.*											
Universalism											
3. He/she thinks it is important that every person in the world be treated equally. He/she believes everyone should have equal opportunities in life.	peqopt	0.7659	1.3875	0.5166	1.4101	-2.0847	0.7417	0.9265	0.387	0.8041	0.6909
8. It is important to him/her to listen to people who are different from him/her. Even when he/she disagrees with them, he/she still wants to understand them.	pudrst	0.7221	1.407	0.3579	1.3312	-0.0054	0.8218	0.9246	0.1818	0.6551	0.6981
19. He/she strongly believes that people should care for nature. Looking after the environment is important to him/her.	penv	0.6762	1.1989	0.1338	1.3178	0.477	0.6712	0.9067	0.142	0.7047	0.6621
Benevolence											
12. It's very important to him/her to help the people around him/her. He/she wants to care for other people.	phlppl	0.7587	1.0513	0.3058	1.38	0.7475	1.0747	1.1601	0.3542	0.9254	0.8359
18. It is important to him/her to be loyal to his/her friends. He/she wants to devote himself/herself to people close to him/her.	plylfr	1.3257	1.6439	1.2661	1.7538	0.9506	1.3168	1.4165	0.5717	1.0708	1.2051
Conformity											
9. He/she believes that people should do what they're told. He/she thinks people should follow rules at all times, even when no-one is watching.	pfrule	-1.0121	-1.7577	-1.6981	-1.3588	-0.6178	-1.8116	0.3025	-0.4083	-0.0155	-0.8252
16. It is important to him/her always to behave properly. He/she wants to avoid doing anything people would say is wrong.	pbhprp	-0.2213	-1.1861	-1.3763	-0.3778	0.0692	-0.7391	0.7636	-0.0522	0.3664	-0.2185
Tradition											
9. It is important to him/her to be humble and modest. He/she tries not to draw attention to himself/herself.	pmodst	-0.168	-0.1426	-1.4549	0.8993	0.0458	0.2929	0.8277	-0.0671	0.7661	0.1695
20. Tradition is important to him/her. He/she tries to follow the customs handed down by his/her religion or his/her family.	ptrad	-0.3046	-1.0215	-0.7038	-0.0096	0.1598	-0.6934	0.4997	-0.0467	0.4556	-0.1279
Security											
5. It is important to him/her to live in secure surroundings. He/she avoids anything that might endanger his/her safety.	psafe	0.4944	-1.4148	-0.8675	0.1303	0.8847	0.1129	1.047	0.2138	0.7954	0.2471
14. It is important to him/her that the government ensures his/her safety against all threats. He/she wants the state to be strong so it can defend its citizens.	pstrgv	0.3639	-0.9659	0.1744	0.5613	0.6307	0.6349	0.8185	0.4312	0.9163	0.4645

Power											
17. It is important to him/her to get respect from others. He/she wants people to do what he/she says.	prspot	-0.2193	-1.0474	-0.2943	-1.1589	-0.0828	-1.566	-0.5098	-0.4668	-1.0769	-0.7369
2. It is important to him/her to be rich. He/she wants to have a lot of money and expensive things.	prich	-1.3769	-2.005	-0.7241	-1.9693	-1.4375	-2.0731	-1.8723	-0.9572	-2.1936	-1.6283
Achievement											
4. It is important to him/her to show his/her abilities. He/she wants people to admire what he/she does.	pshabt	-0.3932	-0.7549	-0.0161	-1.3367	-0.321	-1.1907	-0.9471	-0.2506	-1.5241	-0.796
13. Being very successful is important to him/her. He/she likes to impress other people.	psuces	0.1192	-0.7353	0.39	-0.9676	0.2757	-0.8847	-0.5098	0.0981	-0.9063	-0.3733
Hedonism											
10. Having a good time is important to him/her. He/she likes to "spoil" himself.	pgdtim	0.4279	0.7699	1.0129	-0.1962	0.5352	1.0968	-1.2224	0.2972	0.4773	0.3595
21. He/she seeks every chance he/she can to have fun. It is important to him/her to do things that give him/her pleasure.	pfun	-0.6861	0.1133	0.6126	-1.2645	-0.0764	0.6072	-1.6219	0.059	-0.2294	-0.2945
Stimulation											
6. He/she likes surprises and is always looking for new things to do. He/she thinks it is important to do lots of different things in life.	pdiff	-0.6949	0.7932	0.4655	-0.4486	-0.0319	0.5693	-1.2169	-0.0492	-0.2726	-0.1719
15. He/she looks for adventures and likes to take risks. He/she wants to have an exciting life.	padvnt	-1.8333	0.0129	0.0301	-1.8938	-1.6038	-0.7572	-2.4004	-0.9155	-2.3418	-1.4291
Self-Direction											
1. Thinking up new ideas and being creative is important to him/her. He/she likes to do things in his/her own original way.	pcrtiv	0.377	1.2077	0.8001	0.7905	0.4761	0.5571	0.3225	0.0827	-0.0735	0.3967
11. It is important to him/her to make his/her own decisions about what he/she does. He/she likes to be free to plan and to choose his/her activities for himself.	pfree	0.8787	1.4455	1.0693	1.4074	1.0091	1.2184	0.3849	0.395	0.6965	0.8721
Self-transcendence (mean of universalism+benevolence)		0.85	1.34	0.52	1.44	0.02	0.93	1.07	0.33	0.83	0.82
Self-enhancement (mean of power+achievement)		-0.47	-1.14	-0.16	-1.36	-0.39	-1.43	-0.96	-0.39	-1.43	-0.88
Openness (mean of hedonism+stimulation+self-direction)		-0.26	0.72	0.67	-0.27	0.05	0.55	-0.96	-0.02	-0.29	-0.04
Conservation (mean of conformity+tradition+security)		-0.14	-1.08	-0.99	-0.03	0.2	-0.37	0.71	0.01	0.55	-0.05
Self-transcendence minus self-enhancement		1.32	2.47	0.68	2.8	0.41	2.35	2.03	0.72	2.26	1.7
Openness minus conservation		-0.11	1.81	1.65	-0.24	-0.14	0.92	-1.67	-0.03	-0.84	0

[a] *For presenting the milieus' socioeconomic positions (status), income and education are treated as continuous variables so that the milieu-specific means can be calculated, transformed onto a common scale with a minimum of 0 and a maximum of 1, and then averaged. A value of '1' ('0') indicates the highest (lowest) average score of the milieu members, that is the 5th (1.) income quintile and upper (lower) secondary school.*

[b] *See Schwartz, S. H., Breyer, B., & Danner, D. (2015). Human Values Scale (ESS). Zusammenstellung sozialwissenschaftlicher Items und Skalen (ZIS). doi:https://doi.org/10.6102/zis234*

Source: ESS 8, 2016, n=2470, own calculations

Table S5. Regressions of cohesion factors on milieu membership probabilities and covariates: Trust in cohesion and concerned compliance

	Model 1: Trust				Model 2: Trust				Model 3: Concerned compliance				Model 4: Concerned compliance			
	coef[a]	s.e.[b]	Confidence interv. (90%)		coef	s.e.	Confidence interv. (90%)		coef	s.e.	Confidence interv. (90%)		coef	s.e.	Confidence interv. (90%)	
Intercept	-0.041	0.042	-0.095	0.013	-0.368	0.124	-0.527	-0.209	0.000	0.039	-0.051	0.051	-0.300	0.121	-0.455	-0.145
Milieus																
1	0.159	0.084	0.052	0.267	0.170	0.081	0.065	0.274	-0.004	0.080	-0.107	0.099	0.010	0.087	-0.101	0.120
2	0.176	0.129	0.011	0.341	0.165	0.127	0.003	0.328	0.008	0.115	-0.139	0.156	-0.013	0.107	-0.150	0.124
3	-0.421	0.136	-0.595	-0.246	-0.309	0.157	-0.511	-0.108	-0.424	0.126	-0.585	-0.263	-0.335	0.141	-0.515	-0.154
4	0.107	0.129	-0.058	0.272	0.069	0.134	-0.103	0.240	0.185	0.114	0.039	0.332	0.145	0.106	0.009	0.281
5	0.260	0.176	0.034	0.486	0.233	0.106	0.097	0.368	0.070	0.175	-0.155	0.295	0.024	0.176	-0.201	0.250
6	-0.144	0.147	-0.332	0.045	-0.154	0.178	-0.382	0.074	0.063	0.135	-0.110	0.236	0.025	0.169	-0.191	0.241
7	-0.059	0.145	-0.245	0.127	-0.097	0.160	-0.302	0.108	0.232	0.123	0.075	0.389	0.203	0.114	0.056	0.349
8	-0.319	0.148	-0.508	-0.129	-0.243	0.150	-0.436	-0.051	-0.445	0.175	-0.669	-0.221	-0.314	0.288	-0.683	0.056
9	0.239	0.135	0.066	0.412	0.168	0.117	0.018	0.317	0.315	0.123	0.157	0.472	0.256	0.099	0.129	0.382
Sex: Women					0.050	0.037	0.003	0.098					0.080	0.034	0.037	0.123
Age in years					0.007	0.002	0.004	0.009					0.006	0.002	0.003	0.009
East Germany					-0.031	0.040	-0.082	0.021					-0.047	0.036	-0.094	-0.001
Pseudo-R^2 (squared error)	0.067				0.076				0.089				0.095			

[a] All coefficients are effect coded: Deviation from the mean (=zero for nominal variables)
[b] Robust (Sandwich) standard errors
